# Core microbiota for nutrient digestion remained and ammonia utilization increased after continuous batch culture of rumen microbiota *in vitro*

**DOI:** 10.3389/fmicb.2024.1331977

**Published:** 2024-01-24

**Authors:** Mengyu Liu, Tong Wang, Lu Wang, Hanjie Xiao, Jinhui Li, Chunhui Duan, Lijie Gao, Yueqin Liu, Hui Yan, Yingjie Zhang, Shoukun Ji

**Affiliations:** College of Animal Science and Technology, Hebei Agricultural University, Baoding, China

**Keywords:** core microbiota, continuous batch culture, ammonia utilization, rumen, NH_3_-N utilization efficiency

## Abstract

**Introduction:**

This study aimed to investigate the digestive function, urea utilization ability, and bacterial composition changes in rumen microbiota under high urea (5% urea in diet) over 23 days of continuous batch culture *in vitro*.

**Methods:**

The gas production, dry matter digestibility, and bacterial counts were determined for the continuously batch-cultured rumen fluid (CRF). The changes in fermentation parameters, NH_3_-N utilization efficiency, and microbial taxa were analyzed in CRF and were compared with that of fresh rumen fluid (RF), frozen rumen fluid (FRF, frozen rumen fluid at −80°C for 1 month), and the mixed rumen fluid (MRF, 3/4 RF mixed with 1/4 CRF) with *in vitro* rumen fermentation.

**Results:**

The results showed that the dry matter digestibility remained stable while both the microbial counts and diversity significantly decreased over the 23 days of continuous batch culture. However, the NH_3_-N utilization efficiency of the CRF group was significantly higher than that of RF, FRF, and MRF groups (*p* < 0.05), while five core genera including *Succinivibrio*, *Prevotella*, *Streptococcus*, *F082*, and *Megasphaera* were retained after 23 days of continuous batch culture. The NH_3_-N utilization efficiency was effectively improved after continuous batch culture *in vitro*, and *Streptococcus*, *Succinivibrio*, *Clostridium_sensu_stricto_1*, *p.251.o5*, *Oxalobacter*, *Bacteroidales_UCG.001*, and *p.1088.a5_gut_group* were identified to explain 75.72% of the variation in NH_3_-N utilization efficiency with the RandomForest model.

**Conclusion:**

Thus, core bacterial composition and function retained under high urea (5% urea in diet) over 23 days of continuous batch culture *in vitro,* and bacterial biomarkers for ammonia utilization were illustrated in this study. These findings might provide potential applications in improving the efficiency and safety of non-protein nitrogen utilization in ruminants.

## 1 Introduction

Livestock husbandry plays a pivotal role in human food supply and continues to grow in the population. However, the protein feed shortage brings a heavy burden to livestock raising worldwide (Kim et al., 2019). Developing new protein feedstuffs might be one of the important measures to solve this issue. Non-protein nitrogen could be used by ruminants via rumen microbes, and urea is a kind of widely available and cost-effective protein feed component for ruminants. Upon entering the rumen, with the urease secreted by ureolytic bacteria, urea is rapidly hydrolyzed to ammonia, and the ammonia is further utilized by ammonia-utilizing bacteria and converted to microbial crude protein (MCP), which is a kind of high-quality protein (Fuller and Reeds, 1998; Jin et al., 2018; Patra and Aschenbach, 2018). Previous studies demonstrated that the MCP could provide approximately 80% of the protein requirements for ruminant animals, especially when ruminants were fed with a low-protein diet (Abdoun et al., 2006), and increasing the urea supplementation level from 0 to 2% in the diet also could increase MCP content by 64% in the rumen (Zhang et al., 2016).

However, the feeding level of urea was limited because the hydrolysis of urea was always higher than the microbial utilization of ammonia and thus caused ammonia toxicity, a decrease in dry matter intake (Wang et al., 2016), and digestibility (Kertz, 2010). In ruminants raising, a general adaptation period of 14 to 21 days is required when using the urea in diet because ruminants showed a gradual adaptation to the non-protein nitrogen (Adejoro et al., 2020), and the mechanism was inferred as enhancing the ammonia utilization capacity of ammoniacal nitrogen-utilizing bacteria (Patra and Aschenbach, 2018). Improving the adaptation of rumen microbiota to urea-containing feed might be an efficient strategy to enhance the feeding level of urea.

Rumen microbiota transplantation could change the composition and function of rumen microbiota rapidly (Liu et al., 2019), which might have promising applications in improving the urea adaptation and utilization efficiency, but how to obtain rumen microbiota with efficient urea utilization was the major challenge. Previous studies have shown that rumen simulation systems *in vitro* can maintain rumen microbiota composition and function by continuously adding rumen buffer and fermentation substrates (Muetzel et al., 2009), thus producing rumen microbiota by propagation. However, there is still a lack of information about the composition and function changes during the urea adaptation period in a rumen simulation system *in vitro*. Therefore, we wondered how the core bacterial composition and function, as well as the urea-utilizing efficiency, changed in rumen simulation systems *in vitro* after a long period of urea adaptation higher than 21 days.

Herein, in this study, the rumen microbiota was batch-cultured continuously for 23 days *in vitro*, and we compared the fermentation parameters, urea-utilizing efficiency, and bacterial composition of the continuously batch-cultured rumen fluid with the fresh rumen fluid. As frozen microbiota was usually used in microbiota transplantation (Lee et al., 2016; Kim et al., 2021), and only a portion of rumen fluid was replaced in rumen microbiota transplantation (DePeters and George, 2014; Yin et al., 2021), the bacterial composition and function of frozen rumen fluid (stored at −80°C for 1 month) and mixed rumen fluid (1 of 4 continuously batch-cultured rumen fluid and 3 of 4 fresh rumen fluid) were also assessed. This finding might help improve the security and efficiency of using urea in the diet of ruminants.

## 2 Materials and methods

This research was carried out at the Animal Experimental Center of Hebei Agriculture University from January 2022 to June 2023. The experimental protocol was approved by the Ethical Committee of Hebei Agricultural University (ID: 2021091).

### 2.1 Animals and feed management

The rumen fluid used in this experiment was collected from 6 Hu sheep of approximately 2 years old with permanent rumen fistulas, and the sheep were housed at the Animal Experimental Center of Hebei Agricultural University. The diet was formulated according to NRC2007 (50 kg body weight, 200 g daily gain, the metabolizable energy, and crude protein content in the diet was 11.09 MJ/kg and 12.28% as dry matter basis, respectively). The feed was collected and dried at 65°C for 48 h to achieve air-dried samples, sieved through a 10-mesh sieve, and stored in a dry and sealed environment for nutrient analysis (Table 1).

**Table 1:** Composition and nutrient content of the experimental diet (dry matter basis).

Ingredients, %		Nutrient content, %	
Peanut seedling	14.70	DM	96.56
Maize straw	13.56	ME, MJ/kg	11.09
Corn	56.37	CP	12.28
Soybean Meal	13.16	NDF	32.32
Premix	0.98	ADF	21.57
CaHPO_4_	0.17	Ca	0.80
Talcum powder	0.62	P	0.40
NaCl	0.44		

The premix provided VA 260,000 IU, VD 386,000 IU, VE 600 IU, Cu 0.50 g, Zn 3.60 g, Fe 10.60 g, Mn 3.50 g, Se 14 mg, I 110 mg, and Co 44 mg per kilogram in dry matter basis. Metabolizable energy is calculated based on the ingredient contents, and the other nutrient content was measured. DM, Dry matter; ME, Metabolizable energy; CP, Crude protein; NDF, Neutral detergent fibers; ADF, Acid detergent fibers; Ca, Calcium; P, Phosphorus.

### 2.2 Rumen fluid preparation

#### 2.2.1 Fresh rumen fluid

Fresh rumen digesta was collected through the rumen fistula before morning feeding and was filtered through four layers of sterile gauze. Then, the rumen fluid was collected and put into a thermos flask filled with CO_2_, and it was brought back to the laboratory within 15 min for subsequent experiments.

#### 2.2.2 Frozen rumen fluid

The fresh rumen fluid was collected following the previous statement and stored at −80°C for 1 month before the subsequent experiment.

#### 2.2.3 Continuously batch-cultured rumen fluid

**Figure 1:**
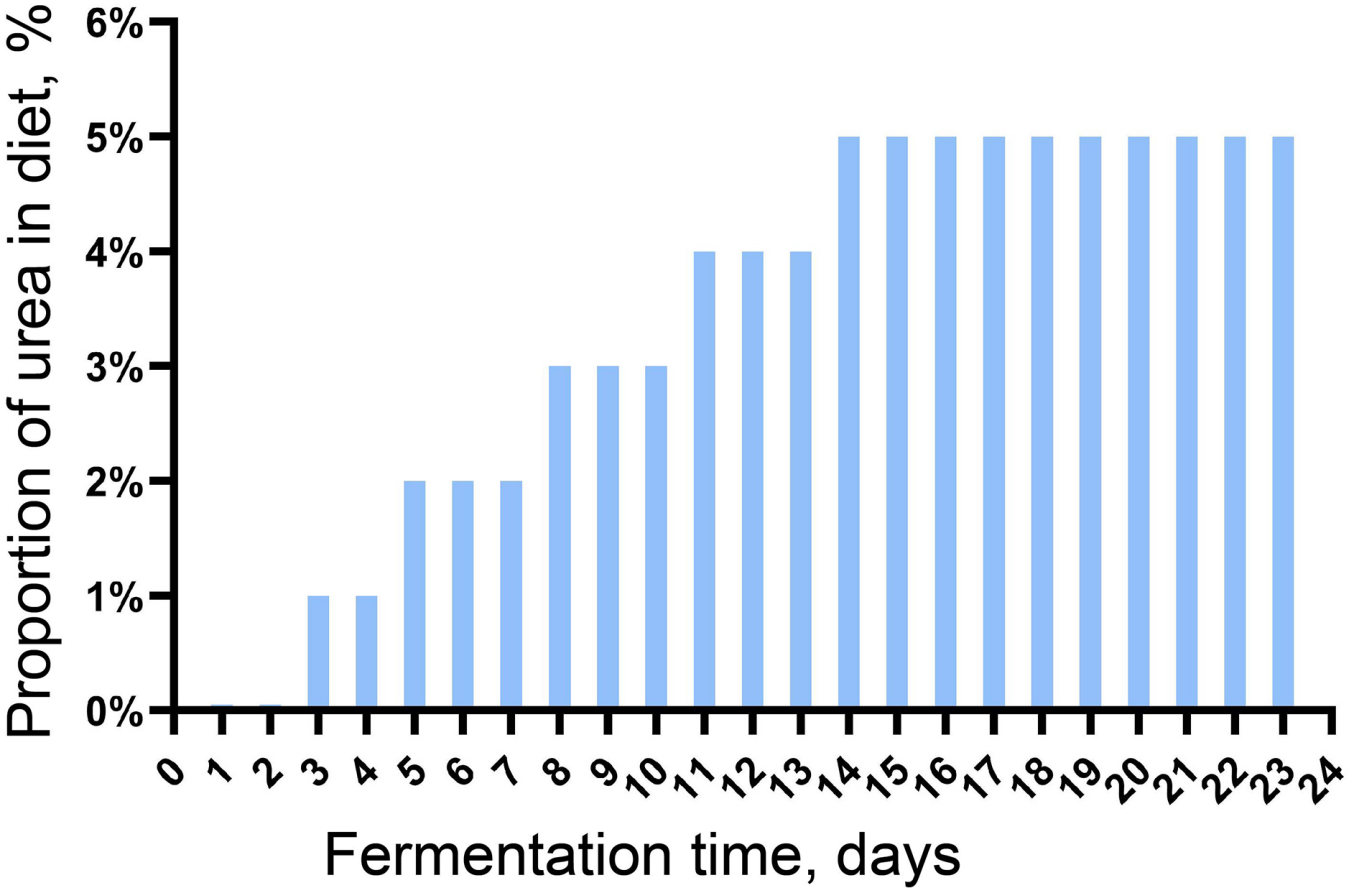
Daily Urea Addition in diet during continuous batch culture *in vitro* (%DM).

The microbiota in the fresh rumen fluid was continuously batch-cultured in an *in vitro* batch culture system for 23 days before the subsequent experiment. The rumen buffer (pH = 6.86) was prepared following the method by Menke et al. (1979), and the batch culture was conducted and modified from our previous study (Li et al., 2022). In brief, the rumen buffer was preheated in the water bath at 39°C for 30 min with CO_2_ to remove oxygen. In total, 30 g of feed containing urea (increased amount gradually, Figure 1) was put into a 5.5 × 4.5 cm nylon bag (300 mesh), and the nylon bag was placed into a 2 L fermentation bag. Then, 200 mL of the preheated rumen buffer and 100 mL of fresh rumen fluid (also preheated at 39°C for 30 min) were mixed and added to the fermentation bag. Four replicates were set up in this trial, and the urea used in this experiment was with purity higher than 99.0%. One round of batch culture lasted for 24 h, and gas production was measured using a graduated syringe. At the end of each round of batch culture, solid and fluid fractions were separated by filtering the digesta through four layers of sterile gauze, and the solid fraction was collected for dry matter digestibility determination; the fluid fraction was collected and mixed with the preheated rumen buffer at the ratio of 1:2 for another round of batch culture. The continuous batch culture *in vitro* was carried out in 23 rounds.

#### 2.2.4 Mixed rumen fluid

Overall, 3 of 4 fresh rumen fluid was mixed with 1 of 4 continuously batch-cultured rumen fluid before the subsequent experiment.

### 2.3 Rumen fermentation with different types of rumen fluid *in vitro*

Four treatment groups were set up, namely, RF group (fresh rumen fluid), FRF group (frozen rumen fluid at −80°C for 1 month, ice water bath for 8 h, and preheated at 39°C for 30 min before used), CRF group (continuously batch-cultured rumen fluid for 23 days), and MRF group (3 of 4 fresh rumen fluid mixed with 1 of 4 continuously batch-cultured rumen fluid).

Before rumen fermentation *in vitro*, the bacterial content in each group was counted. In brief, after being stained with ammonium oxalate-crystal violet and saline multiplicity dilution, the bacteria were counted using a hemocytometer under 1,000x magnification. The bacterial content was calculated as follows: Bacterial Count/ml = N/S*D*16*10*100, where N was the number of counting grid squares, S was the total number of squares counted, and D was the dilution factor. As the bacteria content in different groups was also different, the bacterial content in each group was regulated to 7.45 × 10^9^/ml, with rumen buffer solution for rumen fermentation *in vitro*.

Six replicates were set up for each group, and the rumen fermentation with anaerobic condition was performed following the previous protocol (Li et al., 2022) with some modifications: 3 g of diet containing 5% urea (dry matter basis) was used as fermentation substrate, and the fermentation lasted for 48 h. Gas production was measured using a graduated syringe; 2 mL of fluid was collected at 2, 4, 6, 8, 10, 12, 24, and 48 h, respectively. At the end of the fermentation, the bags were placed in an ice water bath immediately to terminate fermentation; The nylon bag containing feed residues was rinsed three times with distilled water and then dried at 65°C for 48 h; the liquid fraction was also collected and stored at −80°C for determination of pH, MCP, and bacterial composition.

### 2.4 Chemical analysis

The pH of the fluid fraction was determined using an EL20-type acidimeter. The MCP protein was determined following the method described by Makkar et al. (1982), and the concentration of NH_3_-N was measured with the phenol-hypochlorite colorimetric method (Lv et al., 2019). Gas production parameters were calculated using the LELAG mathematical model: GP = b(1–2.718(−c(t-LAG))), where b represented theoretical gas production (ml), c represented gas production rate, and t represented time point (Wang et al., 2011). The diet substrate and undegraded residuals were dried at 65°C for 48 h, and the dry matter (DM) was determined following the AOAC method (AOAC, 2016). The digestibility of dry matter was calculated according to the formula as follows: [(Initial Dry Matter Content–Final Dry Matter Content)/Initial Dry Matter Content] × 100%.

### 2.5 High-throughput sequencing

Total microbial DNA was extracted from the rumen fluid samples after 48 h of rumen fermentation *in vitro* using the E.Z.N.A. Soil DNA Kit (Omega Bio-tek, Inc., United States), and the quality and concentration of the DNA were assessed using the Nanodrop 2000 (Thermo Fisher Scientific, Inc., United States). The V3-V4 regions of the 16S rRNA gene were amplified with the primers 338F (5′-ACTCCTACGGGAGGCAGCAG-3′) and 806R (5′-GGACTACHVGGGTWTCTAAT-3′) using an ABI 9700 PCR instrument (Applied Biosystems, Inc., USA). The PCR amplification system consisted of 1 μL of forward primer (5 μM) and reverse primer (5 μM), 3 μL of DNA template (30 mg total template), 3 μL of BSA (2 ng/μl) and ddH_2_O, and 12.52 μL of Taq Plus Master Mix. The PCR amplification program was as follows: pre-denaturation for 5 min (95°C), denaturation for 45 s (95°C), annealing for 50 s (55°C), extension for 45 s (72°C) for 28 cycles, and extension for 10 min (72°C). The PCR products were purified using the Nucleic Acid Purification Kit (Agencourt AMPure XP, Beckman Coulter, Inc., United States), and finally, high-throughput sequencing was performed on the Illumina Miseq platform (Illumina, Inc., United States), following our previous study (Li et al., 2022).

### 2.6 Bioinformatic analysis

After filtering out the low-quality reads with a quality score lower than 20 over a 50 bp sliding window and sequences with reads shorter than 50 bp with Trimmomatic (v0.36; Gong et al., 2019), the sequencing raw data were assembled using Pear (v0.9.6) software. Vsearch (version 2.0.3) was then used to remove short sequences and chimeras in assembled sequences. Representative sequences were aligned at the Silva138 database with a 97% sequence similarity threshold, to generate operational taxonomic units (OTUs). The α-diversity and β-diversity indexes were analyzed using QIIME (v1.8.0) software.

### 2.7 Statistical analysis

The rumen fermentation parameters after 48 h of rumen fermentation *in vitro,* including DM digestibility, 48 h cumulative GP, theoretical GP, pH, and MCP, were analyzed using one-way ANOVA, and comparison was performed with Duncan’s method using SPSS 26.0 software; the area under the curve (AUC) of dynamic changes in NH_3_-N content in rumen fluid was calculated, changes in rumen microbial community structure were analyzed based on principal component analysis and ANOSIM statistics, and RandomForest analysis was performed with the RandomForest package using R software (v4.1.2) with ntree = 10,000, mtry = p/3, where p is the number of bacterial taxa, and signature bacteria were screened by increasing mean squared error (%IncMSE) and cross-validation. Data were expressed as means and standard error of means (SEM), and *p* < 0.05 was considered significantly different.

## 3 Results

### 3.1 Dynamic changes in gas production and dry matter digestibility during continuous batch culture *in vitro*

**Figure 2:**
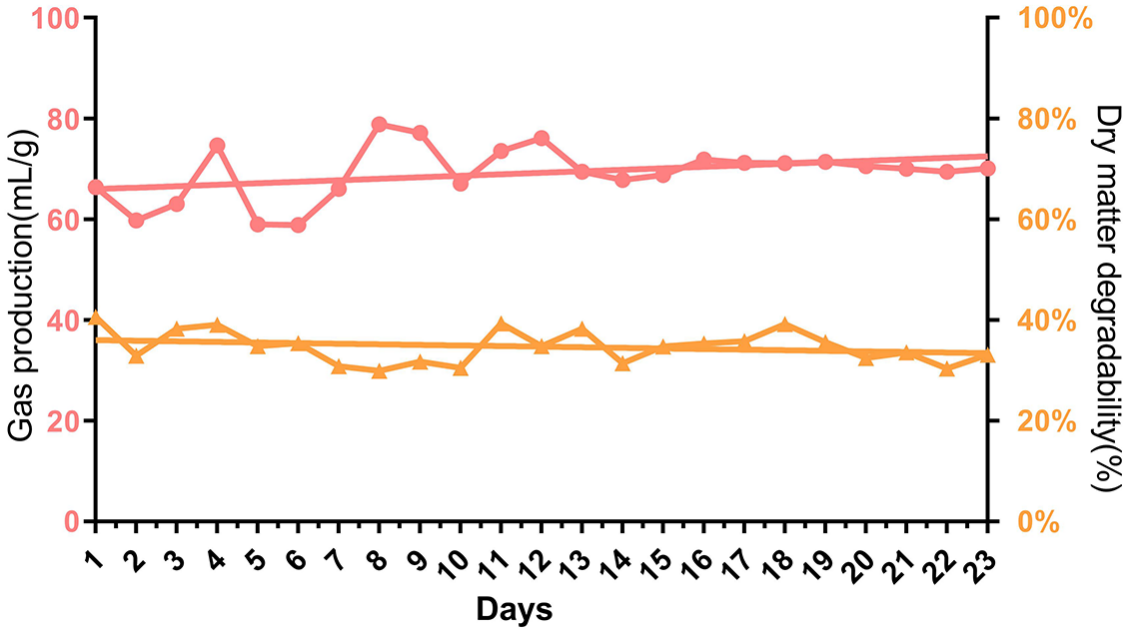
Dynamic changes of gas production and dry matter digestibility during continuous batch culture *in vitro.*

During the first 13 days of gradually increasing urea addition from 0 to 5%, the gas production showed obvious fluctuations from 58.87 mL/g to 77.18 mL/g. While the urea addition was stabilized at 5% after 13 days of fermentation, the gas production became relatively stable at approximately 70.26 mL/g. The dry matter digestibility remained stable with an average of approximately 34.74% over 23 days of continuous rumen batch culture *in vitro* (*p* > 0.05, Figure 2).

### 3.2 Bacterial count of continuous fermentation fluid

After 23 days of continuous batch culture, the bacterial counts in the CRF group were approximately 3.4 × 10^10^, significantly lower than that in fresh rumen fluid (7.54 × 10^9^) but still higher than that in frozen rumen fluid (2.48 × 10^10^; *p* < 0.05; Table 2).

**Table 2:** Bacterial counts in rumen fluid during continuous batch culture *in vitro.*

Items	Groups			SEM	*p*-value
	RF	FRF	CRF		
Bacterial count, *10^9^/mL	34.30^a^	24.80^b^	7.50^c^	13.59	0.00

Values within a row with different superscripts indicate significant differences (*p* < 0.05). RF, Fresh rumen fluid; FRF, Frozen rumen fluid; CRF, Continuously batch-cultured rumen fluid; SEM, Standard error of the mean.

### 3.3 The effects of continuously batch-cultured rumen fluid on fermentation parameters *in vitro*

To confirm the function of the microbial community after continuous rumen batch culture *in vitro*, the fermentation parameters of four groups were compared. The results showed that both the 48 h cumulative and theoretical gas production of the CFR group were significantly lower than those of the RF and MRF groups (*p* < 0.05), but no significant difference was detected in the DM digestibility, pH, and MCP concentration between the groups (*p* > 0.05; Table 3).

**Table 3:** Ruminal fermentation parameters of different groups *in vitro.*

Items	Groups				SEM	*P*-value
	RF	FRF	CRF	MRF		
DM digestibility, %	59.58	59.36	58.54	59.38	0.20	0.30
48 h Cumulative GP, mL/g	143.11^a^	134.67^b^	134.57^b^	143.30^a^	1.21	0.00
Theoretical GP, mL/g	201.69^a^	146.65^c^	149.68^c^	171.28^b^	4.72	0.00
pH	6.83	6.87	6.90	6.85	0.01	0.50
MCP, μg/mL	118.30	115.94	114.55	114.36	0.74	0.35

Values within a row with different superscripts indicate significant differences (*p* < 0.05). DM, Dry Matter; GP, Gas production; MCP, Microbial crude protein; RF, Fresh rumen fluid; FRF, Frozen rumen fluid; CRF, Continuously batch-cultured rumen fluid; MRF, Mixed rumen fluid by 1/4 CRF and 3/4 RF; SEM, Standard error of mean.

### 3.4 The NH_3_-N utilization efficiency of rumen microbiota after continuous rumen batch culture *in vitro*

**Figure 3:**
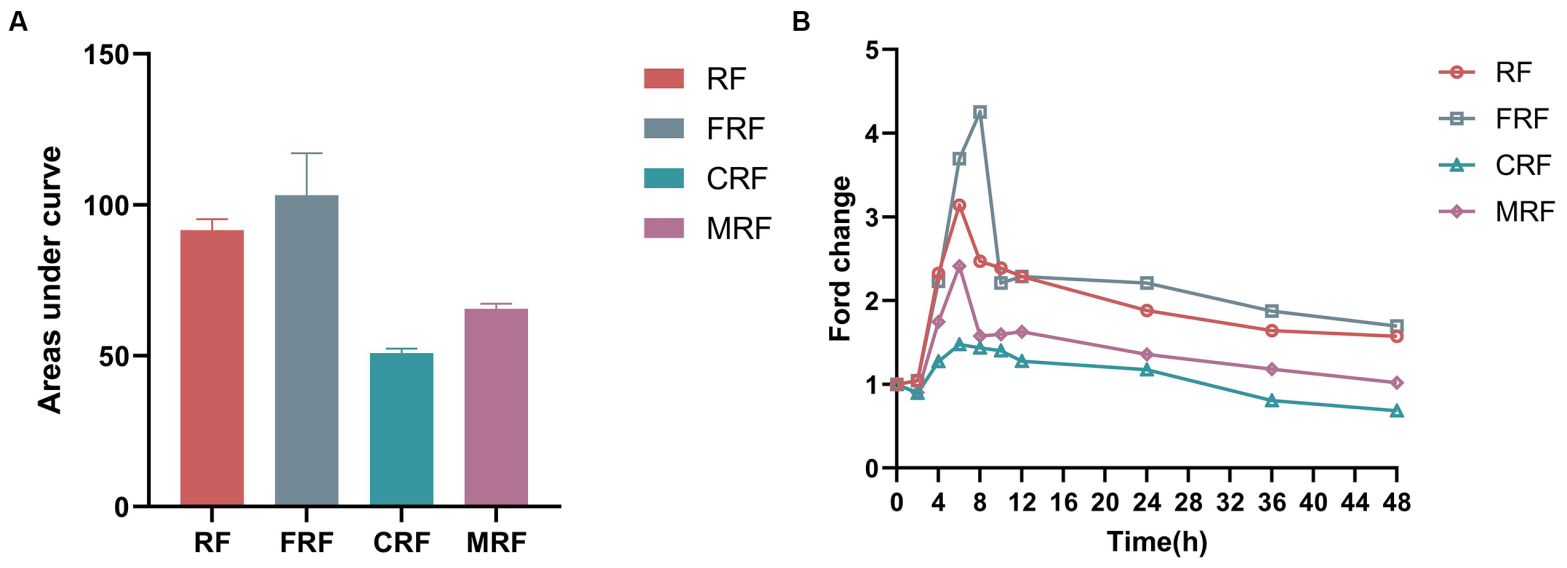
The NH_3_-N utilization efficiency of microbiota after continuous rumen batch culture *in vitro*. **(A)** dynamic change of NH_3_-N content with rumen batch culture *in vitro*; **(B)** the area under the curve of the dynamic change of NH_3_-N content with rumen batch culture *in vitro*. RF, Fresh rumen fluid; FRF, Frozen rumen fluid; CRF, Continuously batch-cultured rumen fluid; MRF, Mixed rumen fluid by 1/4 CRF and 3/4 RF.

As the microbiota of rumen fluid was domesticated after continuous batch culture *in vitro*, we wondered whether the NH_3_-N utilization efficiency would increase. Here, we observed the fold change in NH_3_-N concentrations against its initial concentration at 0 h of fermentation, which significantly differed between the groups after 48 h of *in vitro* fermentation. The highest NH_3_-N concentration in the FRF, RF, and MRF groups was after 6–8 h of batch culture, with NH_3_-N concentrations rising 4.2, 3.1, and 2.4 times, respectively, while that in the CRF group was at 4 h, with NH_3_-N rising approximately 1.5 times (Figure 3A). The area under the curves of dynamic NH_3_-N content in the CRF group was also significantly lower than that in the RF, FRF, and MRF groups (Figure 3B; *p* < 0.05).

### 3.5 Bacterial composition in rumen fluid after batch culture *in vitro*

**Figure 4:**
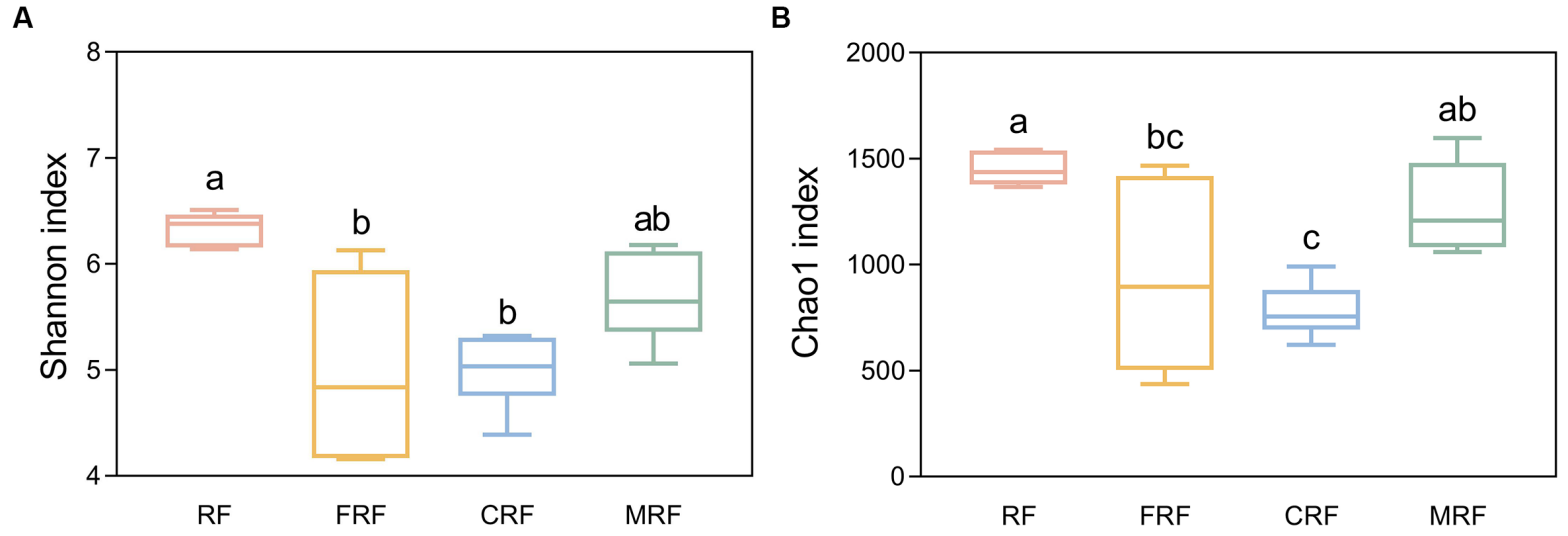
Comparing the alpha diversity index between groups. **(A)** shannon index between groups; **(B)** Chao1 index between groups. RF, Fresh rumen fluid; FRF, Frozen rumen fluid; CRF, Continuously batch-cultured rumen fluid; MRF, Mixed rumen fluid by 1/4 CRF and 3/4 RF.

**Figure 5:**
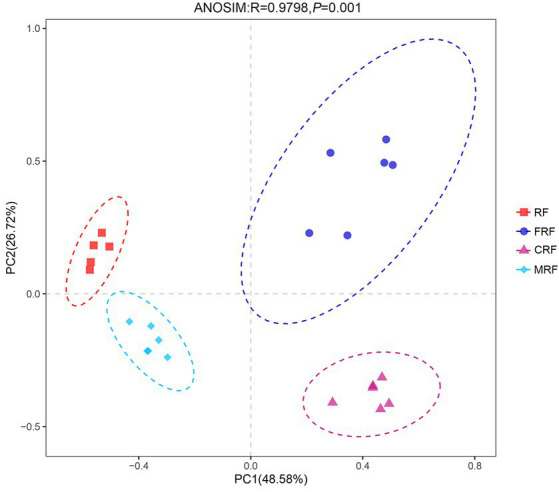
PCoA analysis based on Bray–Curtis distance at OTU level. RF, Fresh rumen fluid; FRF, Frozen rumen fluid; CRF, Continuously batch-cultured rumen fluid; MRF: Mixed rumen fluid by 1/4 CRF and 3/4 RF.

With high-throughput sequencing of 16S rRNA genes, 29 phyla, 62 classes, 130 orders, 205 families, and 337 genera were identified. Both Chao1 and Shannon indexes of the CRF group and FRF group showed significantly lower compared with that of the RF group (*p* < 0.05; Figure 4). A significant difference in the microbial community structure among the RF, FRF, CRF, and MRF groups with PCoA analysis was also observed (*R* = 0.9798; ANOSIM, *p* = 0.001; Figure 5). The PC1 axis explained 48.48% of the variance, while the PC2 axis explained 26.72% of the variance of microbial community structure (Figure 5). This finding suggested that continuous batch culture or frozen treatment could lead to a reduction in microbial diversity.

At the phylum level, Bacteroidota, Firmicutes, and Proteobacteria were the top three dominant phyla (relative abundances higher than 5%); however, the relative abundances of the dominant phyla differed between groups (*p* < 0.05). Bacteroidota were the most dominant phylum in the RF group and MRF group, accounting for 60.21 and 60.78%, respectively, which were significantly higher than that of the FRF and CRF groups (33.54 and 24.97%, *P*<0.05). Firmicutes were the second most abundant phylum, on average, and it was the most dominant phylum in the FRF group and CRF group, accounting for 51.03 and 37.53%, respectively, which were significantly higher than that in the RF group and MRF group (25.37 and 14.98%, *P*<0.05). Proteobacteria had the highest abundance in the CRF group, accounting for 33.81%, which was significantly higher than that in the other three groups (*p* < 0.05), with 9.7, 11.48, and 20.6%, respectively. In addition, compared with the RF group, Spirochaetota and Verrucomicrobiota showed a significant decrease in the abundance in the CRF group (*P*<0.05; Table 4).

**Table 4:** The relative abundance of bacterial composition in different groups at phylum level (%).

Phylum	Groups				SEM	*P*-value
	RF	FRF	CRF	MRF		
Bacteroidota	60.21^a^	33.54^b^	24.97^b^	60.78^a^	3.73	0.00
Firmicutes	25.37^c^	51.03^a^	37.53^b^	14.98^d^	3.29	0.00
Proteobacteria	9.70^c^	11.48^c^	33.81^a^	20.16^b^	2.39	0.00
Spirochaetota	1.82^a^	1.11^ab^	0.84^b^	1.00^ab^	0.15	0.14
Verrucomicrobiota	1.16^a^	0.10^c^	0.12^b^	0.78^a^	0.12	0.00

Values within a row with different superscripts indicate significant differences (*p* < 0.05). RF, Fresh rumen fluid; FRF, Frozen rumen fluid; CRF, Continuously batch-cultured rumen fluid; MRF, Mixed rumen fluid by 1/4 CRF and 3/4 RF; SEM, Standard error of the mean.

At the genus level, *Prevotella*, *Succinivibrio*, *F082*, and *Rikenellaceae_RC9_gut_group* were the dominant genera with relative abundances higher than 5% in both of the RF and MRF groups; For the FRF group, *Streptococcus*, *Bacteroides*, *Megasphaera*, *Prevotella*, *Succinivibrio*, and *Rikenellaceae_RC9_gut_group* were the dominant genera. For the CRF group, *Succinivibrio, Prevotella,* and *Megasphaera* were the dominant genera. The relative abundances of *Prevotella*, *F082*, and *Rikenellaceae_RC9_gut_group* in the CRF group were significantly lower than that in the RF and MRF groups (*p* < 0.05). In addition, the abundance of *Succinivibrio* in the CRF group was significantly higher than that in the RF and MRF groups by 5.4 and 1.9 times, respectively (*p* < 0.05); the relative abundance of *Megasphaera* in the CRF group was significantly higher than that in the RF and MFR groups by 25.5 and 9.1 times, respectively (*p* < 0.05; Table 5).

**Table 5:** Relative abundance of bacterial composition in different groups at the genus level (%).

Genus	Groups				SEM	*P*-value
	RF	FRF	CRF	MRF		
*Prevotella*	24.41^a^	9.17^b^	8.81^b^	23.20^a^	1.86	0.00
*Succinivibrio*	6.13^c^	7.55^c^	33.21^a^	17.60^b^	2.54	0.00
F082	15.16^b^	0.58^c^	1.49^c^	20.85^a^	1.99	0.00
*Rikenellaceae*_RC9_gut_group	13.11^a^	5.12^b^	1.99^b^	12.02^a^	1.11	0.00
*Streptococcus*	4.92^b^	25.96^a^	0.35^b^	0.64^b^	2.50	0.00
*Megasphaera*	0.31^b^	10.42^a^	7.90^a^	0.87^b^	1.22	0.00
*Bacteroides*	0.07^b^	11.97^a^	0.77^b^	0.15^b^	1.21	0.00

Values within a row with different superscripts indicate significant differences (*p* < 0.05). RF, Fresh rumen fluid; FRF, Frozen rumen fluid; CRF, Continuously batch-cultured rumen fluid; MRF, Mixed rumen fluid by 1/4 CRF and 3/4 RF; SEM, Standard error of the mean.

### 3.6 Core bacteria in rumen fluid after batch culture *in vitro*

**Figure 6:**
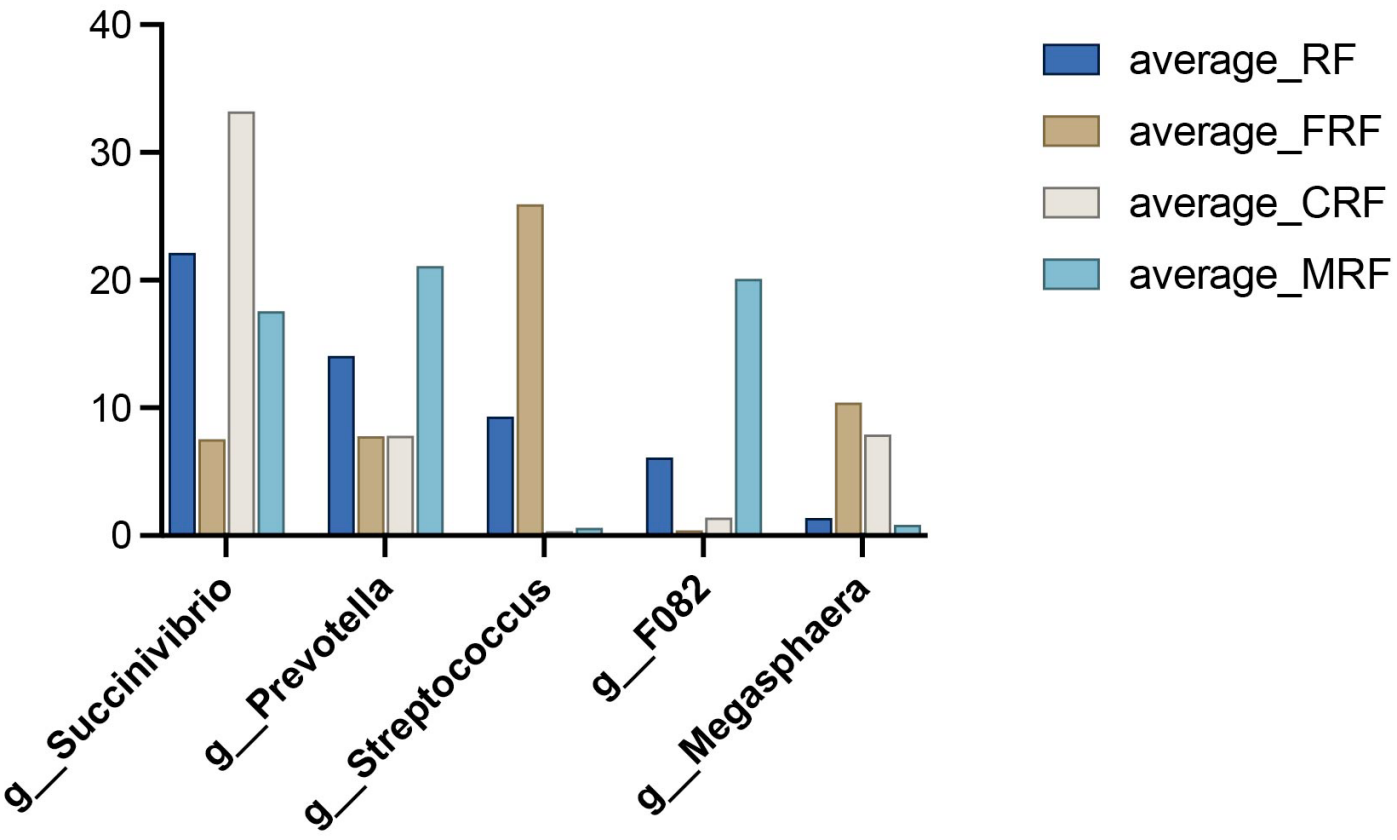
Relative abundance of core bacteria between groups. RF, Fresh rumen fluid; FRF, Frozen rumen fluid; CRF: Continuously batch-cultured rumen fluid; MRF, Mixed rumen fluid by 1/4 CRF and 3/4 RF.

The core bacteria refer to the bacteria that exist in over 80% of all the samples. We observed that 155 OTUs out of the total 2,261 OTUs were core bacteria in all samples, and these core OTUs accounted for 77.5, 86.9, 90.1, and 85.0% in the abundance in the RF, FRF, CRF, and MRF groups, respectively. Moreover, the core bacteria that were identified mainly belonged to the five dominant genera, namely, *Succinivibrio*, *Prevotella*, *Streptococcus*, *F082*, and *Megasphaera*, whose average abundances among all samples were 20.1, 12.7, 9.1, 7.0, and 5.1%, respectively. In the CRF group, the core bacteria of *Succinivibrio*, *Prevotella*, and *Megasphaera* were effectively retained after 23 days of continuous batch culture, and the abundance of *Succinivibrio* was significantly higher than that in the other three groups (*p* < 0.05; Figure 6).

### 3.7 Prediction of bacterial taxa associated with the NH_3_-N utilization efficiency

**Figure 7:**
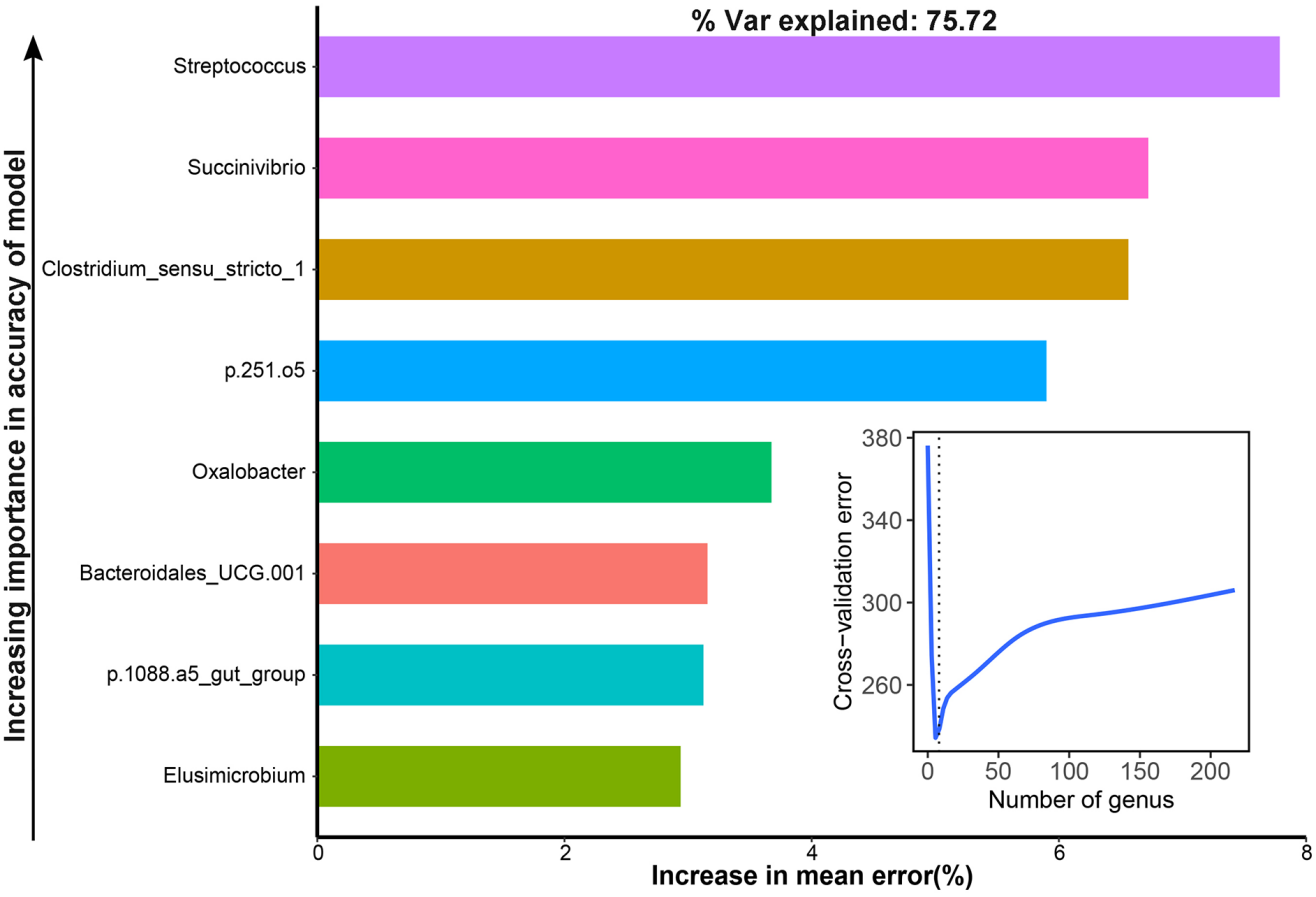
Bacteria associated with the area under the curve (AUC) of dynamic changes of the NH_3_-N content with the RandomForest model.

The area under the curve (AUC) was used to identify bacterial taxa that were associated with the NH_3_-N utilization efficiency. We observed that eight genera were highly associated with the AUC of dynamic changes in NH_3_-N content by cross-validating the error curves, including *Streptococcus*, *Succinivibrio*, *Clostridium_sensu_stricto_1*, *p.251.o5*, *Oxalobacter*, *Bacteroidales_UCG.001*, *p.1088.a5_gut_group,* and *Elusimicrobium*, which could explain 75.72% of the variation of the AUC of dynamic change in NH_3_-N content in different groups (Figure 7).

## 4 Discussion

Gas production and dry matter digestibility are important indicators, reflecting the function of rumen (Pashaei et al., 2010; Zhang M. et al., 2022). Previous studies reported controversial effects of urea supplementation on gas production and dry matter digestibility (Salem et al., 2013; Spanghero et al., 2018; Muthia et al., 2021; Kour et al., 2023), and we detected that bacterial counts decreased but gas production and dry matter digestibility were stable over a 23-day continuous batch culture of rumen microbiota under high urea condition. The theory of functional redundancy in rumen microbiota might support these observations (Wemheuer et al., 2020; Sasson et al., 2022), and we inferred that functional redundant microbes might be partially removed, while core bacteria associated with dry matter digestion might be preserved after 23 days of continuous batch culture.

We then looked into the changes in rumen microbiota after 23 days of continuous batch culture. We observed that microbial diversity significantly changed, but the majority of the removed microbes were non-core members, increasing the proportion of core bacteria. At the phylum level, Bacteroidota, Firmicutes, and Proteobacteria were the dominant phyla in all groups, which was consistent in previous studies (Li et al., 2020). At the genus level, the core bacteria were found mainly in five genera, namely, *Succinivibrio*, *Prevotella*, *Streptococcus*, *F082*, and *Megasphaera*. Among them, *Succinivibrio* was related to the utilization of soluble sugars to produce lactate, acetate, formate, and propionate (Li et al., 2020; Wang Y. et al., 2021; Wang Z. et al., 2021); *Prevotella* is a dominant rumen genus that plays important roles in nitrogen metabolism (Henderson et al., 2015) and hemicellulose degradation(Mizrahi et al., 2021); *Streptococcus*, as one of the dominant genera, belongs to acid-tolerant bacteria (Khafipour et al., 2009) and is negatively correlated with cellulose degradation (Chuang et al., 2020); *F082* was found to be a core genus in rumen fluid and was sensitive to feed components (Hinsu et al., 2021; Choi et al., 2022), it has been reported that *F082* is positively correlated with the propionate production (Ma et al., 2020); *Megasphaera* was often accompanied with *Sharpea* spp., which can increase butyrate production (Kamke et al., 2016). These findings indicated that bacterial composition might be significantly affected after continuous batch culture of rumen microbiota, and the core bacteria retained.

The nitrogen utilization efficiency of rumen microbiota can be directly reflected by the concentration of NH_3_-N in rumen fluid (Patra and Aschenbach, 2018; Guo et al., 2022), and it has been shown that the addition of non-protein nitrogen to the diet significantly increased the NH_3_-N concentration in the rumen (Xu et al., 2019). Microbiota in the rumen was able to fully utilize NH_3_-N to synthesize MCP when NH_3_-N concentrations ranged from 6.3 to 27.5 mg/dL (Satter and Slyter, 1974; Murphy and Kennelly, 1987). In the current study, when 5% urea was added to the diet, the NH_3_-N concentrations of all groups were higher than the maximum suitable concentration (27.5 mg/dL), but a significant reduction in ammoniacal nitrogen was found after continuous batch culture of rumen microbiota, which might be caused by the adaptive evolution of microbial communities in the rumen after continuous batch culture of rumen microbiota under high urea condition. Additionally, we also observed that the nitrogen utilization efficiency of the mixture of fresh rumen fluid and continuously batch-cultured rumen fluid (3 of 4 fresh rumen fluid mixed with 1 of 4 continuously batch-cultured rumen fluid) was also enhanced, which indicated that the nitrogen utilization efficiency might also be enhanced by rumen microbiota transplantation with only a portion of rumen fluid was replaced in the rumen (DePeters and George, 2014; Yin et al., 2021).

We then looked into the core bacteria potentially associated with the NH_3_-N utilization efficiency. After 23 days of continuous batch culture, the urea treatment led to an increase in the abundance of Proteobacteria and a decrease in the abundance of Bacteroidota, which is consistent with the findings of previous studies by adding 0.5% urea (Jin et al., 2016). Proteobacteria exhibited the highest abundance after continuous batch culture under high urea concentration, which is always found in a disrupted rumen microbial ecology or an unstable gut microbial community structure (Shin et al., 2015), and this might be a limitation of the current *in vitro* continuous fermentation system; further research is still needed on the safety of applying artificially domesticated microbial populations. With the RandomForest model, we observed that *Streptococcus*, *Succinivibrio*, *Clostridium_sensu_stricto_1*, *p.251.o5*, *Oxalobacter*, *Bacteroidales_UCG.001*, and *p.1088.a5_gut_group* explained 75.72% of the variation in NH_3_-N utilization efficiency. Strains from *Succinivibrio* and *Streptococcus* could produce urease that catabolizes metabolized urea (Sissons and Hancock, 1993; Zotta et al., 2008; Pope et al., 2011), and *Succinivibrio* exhibited higher abundance in urea-added fermenters (Jin et al., 2016) and positively correlated with ammonia levels in the rumen (Zhang Y. et al., 2022). *Clostridium_sensu_stricto_1* was also related to nitrogen utilization (Zhou et al., 2021) and positively correlated with ammonia concentration in the rumen (Chen et al., 2023; Zhao et al., 2023), which could convert nitrate into ammonia (Chen et al., 2023). There is also evidence that *Clostridium_sensu_stricto_1* is positively correlated with a high-protein diet (Fan et al., 2017), indicating its possible involvement in protein degradation. The strains from *p.251.o5* and *Bacteroidales_UCG.001* had not been successfully cultured (Mu et al., 2022), and their functions were still unclear. *Oxalobacter* was found to be associated with oxalate metabolism (Stewart et al., 2004). The *p.1088.a5_gut_group* shows higher abundance in the rumen of goats with high ammonia nitrogen utilization efficiency (Li et al., 2019), indicating its potential role in ammonia utilization. *Elusimicrobium* is a type of ultramicrobacteria that can use nitrogen with group IV nitrogenase (Zheng et al., 2016; Shelomi et al., 2019). Thus, specific taxa have been found to contribute to the enhancement of NH_3_-N utilization efficiency in the rumen.

## 5 Conclusion

After continuous batch culture of rumen microbiota under high urea conditions, the core functions and bacterial taxa were retained, and the NH_3_-N utilization efficiency increased. *Streptococcus*, *Succinivibrio*, *Clostridium_sensu_stricto_1*, *p.251.o5*, *Oxalobacter*, *Bacteroidales_UCG.001*, and *p.1088.a5_gut_group* had been identified to contribute to the enhancement of NH_3_-N utilization efficiency. These findings indicated that we could achieve rumen microbiota with remaining core bacteria and function and increase NH_3_-N utilization efficiency by continuous batch culture of rumen microbiota *in vitro,* which would provide potential applications in improving the efficiency and safety of non-protein nitrogen utilization in ruminants by rumen microbiota transplantation or manipulating the bacterial composition.

## Data availability statement

The 16S rRNA high-throughput sequencing datasets for this study can be found in the China National Center for Bioinformation, accession number CRA013223.

## Author contributions

ML: Data curation, Formal analysis, Methodology, Writing – original draft. TW: Data curation, Formal analysis, Methodology, Writing – review & editing. LW: Writing – review & editing. HX: Formal analysis, Software, Writing – review & editing. JL: Formal analysis, Methodology, Writing – review & editing. CD: Investigation, Writing – review & editing. LG: Resources, Writing – review & editing. YL: Resources, Writing – review & editing. HY: Project administration, Supervision, Writing – original draft, Writing – review & editing. YZ: Resources, Writing – review & editing. SJ: Conceptualization, Funding acquisition, Supervision, Writing – review & editing.
